# Intervention strategies in early childhood to prevent celiac disease—a mini-review

**DOI:** 10.3389/fimmu.2023.1106564

**Published:** 2023-02-22

**Authors:** Carin Andrén Aronsson, Daniel Agardh

**Affiliations:** Department of Clinical Sciences, Lund University, Malmö, Sweden

**Keywords:** prevention, celiac disease, children, gluten, RCT

## Abstract

A higher intake of gluten during childhood is associated with increased risk of celiac disease, and the incidence of celiac disease peaks shortly after the time point when associations with higher gluten intake during the second and third year of life occur. Additional environmental factors are most likely necessary for celiac disease to develop. It is hypothesized that gastrointestinal infections increase gut permeability and exposure to gluten. Alternatively, infections may lead to gut dysbiosis and chronic inflammation, with leakage of self-antigens that mimic gluten peptides that leads to an autoimmune-like response. Different gluten interventions to prevent celiac disease have been proposed. Early clinical studies suggested an optimal time point introducing gluten between 4 and 6 months of age while the infant is being breastfed. However, later clinical trials on reduced gluten intake given to infants have shown no protection from celiac disease if gluten introduction was delayed or if gluten was introduced in small amounts during the child’s first year of life. Still, more randomized clinical trials (RCTs) are warranted to answer the question if a reduced amount of gluten, not only at the time of introduction during infancy but also in a longer time frame, will prevent children at genetic risk from having lifelong celiac disease. It needs to be clarified whether dietary interventions are effective strategies to be proposed as future prevention of celiac disease in the general population. The present mini-review provides an overview of ongoing or completed RCTs that have focused on interventions during early childhood with the aim of preventing celiac disease.

## Introduction

Celiac disease is a chronic immune-mediated disorder caused by a T-cell-mediated response against gliadin peptides found in gluten-containing grains (1). A characteristic feature of celiac disease is an intestinal mucosa showing villous atrophy on a gluten-containing diet (2), which will result in the restoration of the intestinal mucosa structure after treatment with a gluten-free diet (GFD). Celiac disease is a lifelong condition currently without cure and will therefore lead to relapse of the disease when gluten is reintroduced to the diet even years after GFD (3).

The global prevalence of celiac disease is estimated to be approximately 1% but estimated as high as 3% in Sweden (4). The prevalence of celiac disease is higher in countries with high consumption of wheat (5). Despite that gluten is essential for celiac disease to develop, celiac disease only affects individuals with a genetic predisposition of carrying the human leukocyte antigen (HLA)-risk haplotypes. More than 95% of celiac disease patients carry the HLA-DQA1*05:01-DQB1*02:01 (abbreviated DQ2) and 5% DQA1*03:01-DQB1*03:02 (abbreviated DQ8) haplotypes (1). Moreover, there is a gene dose effect of HLA demonstrating that children with two copies of HLA-DQ2 are at the highest risk of celiac disease and at an early age (6–8). Prospective birth cohort studies have found that 20% of the children carrying the DQ2 haplotypes followed from birth develop celiac disease by the age of 10 years (6). Albeit gluten is considered to be driving a higher prevalence of celiac disease in populations with higher frequencies of HLA-risk genotypes, additional environmental factors such as birth mode, season of birth, viral infections, and altered microbiome may facilitate and/or trigger the immune response to dietary gluten (9–11). Celiac disease can develop at any time over a life span, although the incidence peaks in early childhood (12).

A lot of studies have focused on gluten infant feeding since the steep increase in incidence of celiac disease that affected children younger than age 2 years in Sweden after change in gluten infant feeding recommendations occurred in the mid-1980s, which dropped again a decade later (13). This so-called celiac epidemic co-occurred when the gluten content was changed in the baby formulas and the breastfeeding recommendations were revised, indicating that there could be a window of opportunity to induce tolerance to gluten in young children. Although there is no consensus when is the optimal time point of introducing gluten in the diet, the European Society for Pediatric Gastroenterology, Hepatology, Nutrition (ESPGHAN) recommends gluten to be introduced to the infant’s diet anytime between 4 and 12 months of age, and large gluten amounts during the first months of introduction should be avoided (14). More recent birth cohort studies following children at genetic risk demonstrate that the incidence of celiac disease is highest between 2 and 3 years of life (15, 16) and found an association between gluten intake and celiac disease both in the general population (17) and in genetically at-risk populations (15, 16). However, it has not yet been confirmed whether a reduced intake of gluten amounts will prevent or delay the diagnosis of celiac disease, since many clinical trials performed so far have been underpowered to prove causality (18, 19). Previous primary prevention studies focusing on early infant feeding practices have shown no protection of celiac disease if gluten introduction was delayed (20) or introduced in small amounts during the child’s first year of life (19). The aim of the present mini-review was to provide an overview of completed and ongoing randomized clinical trials with focus on dietary interventions to prevent celiac disease.

## Clinical trials on preventing celiac disease

MEDLINE (PubMed) and Clinical Trials (www.clinicaltrials.gov) were searched for randomized RCTs investigating the potential association between feeding practices and celiac disease, from inception up until October 2022. All completed RCTs investigated dietary interventions with focus on early tolerization to gluten consumption or the effect of timing on gluten introduction. For the current review, five completed and two ongoing trials were included (**Table 1**).

**Table 1:** Description of primary prevention studies focusing on infant feeding practices.

Study/first author [Ref]	Population	Type of intervention	Participants (n)	Country	Duration of study (follow-up)	Results
Completed clinical trials						
BABYDIET/Hummel (21), Beyerlein (22)	Infants with known genetic risk	Gluten introduction at 6 vs. 12 months	150 (control group n=77)	Germany	Up to 3 and 8 years of age	No effect
Selitto (18)	Infants with an FDR-CD	Gluten introduction at 6 vs. 12 months.	30 (control group n=17)	USA	Up to 2 years of age	Delayed gluten introduction reduced the risk of CDA
CELIPREV/Lionetti (20)	Infants with an FDR-CD	Gluten introduction at 6 vs. 12 months	707 (control group n=379)	Italy	Up to 5 years of age	Later gluten introduction of gluten was associated with delayed onset of disease.
Prevent-CD/Vriezinga (19)	Infants with known genetic risk and with an FDR-CD	Small amounts of gluten introduced during 4–6 months of age, with a stepwise and fixed gluten increase until 10 months of age and unrestricted intake thereafter	944 (control group n=480)	Croatia, Germany, Hungary, Israel, Italy, Netherlands, Poland, Spain	Up to 3 years of age	No effect
EAT/Logan (23)	Infants with unknown genetic risk	Introduction to gluten (wheat) from the age of 4 months vs. exclusive breastfeeding until 6 months	1,004 (control group n=516)	England, Wales	Up to 3 years of age	Early introduction reduced the risk of celiac disease
**Ongoing clinical trials**						
PreCiSe	Infants with known genetic risk	Introduction to gluten from the age of 3 years vs. no dietary intervention with/without daily probiotic supplementation	Recruiting	Sweden	Up to 7 years of age	–
GRAIn	Infants with known genetic risk	Follow a gluten reduced diet (<2 g/day) from the age of 6 months or no dietary intervention	Recruiting	Sweden	Up to 10 years of age	–

FDR-CD, first-degree relative (mother, father, or sibling) with celiac disease.

### Completed clinical trials

BABYDIET (ClinicalTrials.gov Identifier: NCT01115621) is a German study enrolling infants carrying HLA genotypes associated with both type 1 diabetes and celiac disease. Children (n=150) were randomly assigned to be introduced to gluten at the age of 6 months (control group) or to delay the introduction of gluten until the age of 12 months. Children were followed for 3 (21) and 8 years (22), respectively. Delaying gluten exposure until the age of 12 months did not reduce the risk for celiac disease autoimmunity (CDA), i.e., persistence of tissue transglutaminase autoantibody (tTGA) positivity, in genetically at-risk children (22).

Another randomized double-blind study by Selitto et al. (18), conducted on 30 US infants born between 2005 and 2009, with a first-degree relative (FDR) with biopsy-proven celiac disease, were enrolled to a dietary intervention with two arms. Enrolled participants were asked to keep a gluten-free diet (GFD) until 12 months of age (i.e., late introduction) or to introduce gluten at 6 months (i.e., early introduction). Participants randomized to early introduction of gluten were instructed to continue exclusive breastfeeding in combination with daily supplementation of a purified gluten (3 g at 6–9 months and 5 g at 9–12 months). The small group of children were followed until the age of 24 months, and the study concluded that delayed gluten introduction (6 vs. 12 months) lead to a lower incidence of CDA. The results suggest that a delayed introduction of gluten in the diet of genetically susceptible infants may delay the onset of celiac disease.

CELIPREV (ClinicalTrials.gov Identifier: NCT00639444) is an Italian multicenter study that tested if introduction to gluten at 6 months compared with that at 12 months would reduce the risk of celiac disease (20). The primary outcome was celiac disease diagnosed at the age of 5 years. At 2 years of age, children introduced to gluten at 6 months had significantly higher proportion of CDA and celiac disease compared with children introduced at 12 months of age. At 5 years of age, the between-group differences were no longer significant for CDA or celiac disease. The conclusion was that later introduction was only associated with a delayed onset of celiac disease.

Prevent-CD (Clinical Trial Identifier ISRCTN74582487) a multinational double-blind placebo-controlled study, based on infants with an FDR with celiac disease, tested the hypothesis of possible induction of tolerance to gluten in at-risk children by introducing small amounts of gluten (100 mg gluten/day) during early infancy (at the age of 4–6 months) (19). Compared with placebo, small amounts of gluten during early infancy did not reduce the risk of celiac disease by the age of 3 years.

The EAT study (Enquiring About Tolerance) (Clinical Trial Identifier ISRCTN14254740) is studying children from the general population in England/Wales, randomized to consume allergenic foods from the age of 4 months compared with exclusive breastfeeding until 6 months (23). In the general population, the introduction to gluten (high-dose consumption, 500 mg/day) was associated with reduced prevalence of celiac disease.

### Ongoing clinical trials

The Prevention Celiaki i Skåne (PreCiSe) (ClinicalTrials.gov Identifier NCT03562221) study is currently enrolling HLA-DQ2/DQ2 infants genotyped at birth and invited from the general population in the south of Sweden. The aim is to investigate if a GFD during the child’s first 3 years of life prevents celiac disease up to 7 years of age compared with no dietary intervention. Another aim is to investigate if a daily intake of a probiotic supplement during the child’s first 3 years of life prevents celiac disease up to 7 years of age compared to the groups keeping a GFD or no dietary intervention, respectively. The PreCiSe study aims to enroll 300 infants with a baseline visit before the age of 4 months (before 17 weeks of age). Participants visit the clinic every 3 months for the first 3 years of life and annually thereafter until 7 years of age. The study product is double blinded, but the active product contains two strains (*Lactobacillus plantarum* HEAL9 and *Lactobacillus paracasei* 8700:2), at a total dose of 1×10^10^ cfu/sachet. The strains are chosen due to their documented effect of the intestinal environment and different physiological effects, i.e., *L. plantarum* HEAL9 is targeting the permeability of the mucosa and *L. paracasei* 8700:2 is targeting the immune system by stimulating regulatory T cells (24–28).

Study data are collected through interviews with the caregivers (29), and longitudinal data about environmental factors are collected. All enrolled participants complete 3-day food records in conjunction with clinic visits every 3 months. In addition, children allocated to the dietary intervention are instructed to delay time to introduction of gluten-containing cereals (wheat, rye, and barley) until the age of 3 years and during the first 3 years of life following a GFD. Families will be provided with gluten-free foods (flour, pasta, crisp bread, and porridges) and have counseling with a registered dietitian at 6 months of age and then annually. In addition, families will have ongoing access to the dietitian who will support with gluten-free recipes and issue certificate for GFD for daycare/pre-school. Compliance to the GFD will be monitored, and overall dietary adherence will be checked annually. Serum samples are collected every 3 months for the first 3 years and then annually up to the age of 7 years and analyzed for tTGA using radio binding assays. Stool and saliva samples are collected every 3 months during the intervention phase to be used for microbiome and metabolomic analyses. The primary study outcome is CDA, and the secondary outcome is biopsy-proven celiac disease.

The Gluten Reduction After Infancy and Risk of Celiac Disease (GRAIn) study **(**ClinicalTrials.gov Identifier: NCT04593888) is currently enrolling Swedish infants carrying the following HLA genotypes: DR3/DR4-DQ8, DR3/DR4-DQ7, and DR3/X. Children carrying any of these genotypes are estimated to have a moderate (10–15%) increased risk of developing celiac disease before the 10 years compared to the general population. The objectives are to investigate whether a gluten-restricted diet during the first 5 years of life reduce the risk of CDA and onset of celiac disease up to the age of 10. The GRAIN study aims to enroll 1,000 HLA eligible infants to the study by the age of 6 months, to be randomized to one of two study arms (no dietary intervention or to follow a gluten-reduced diet containing <2 g/gluten per day), and followed with intervention visits every 6 months until the age of 5 years and with annual visits for the follow-up period until the age of 10 years. Study data are collected through interviews with the caregivers, and information about environmental factors is collected. Information about gluten consumption is estimated using 3-day food records, collected in conjunction with the clinical visits. Throughout the study, a registered dietitian will provide specific dietary advice to the caregivers of children in addition to offering regular contact with participants (i.e., the parents) randomized to the gluten-restricted group. Compliance to the gluten-reduced diet and overall dietary adherence are monitored. The gluten restricted diet shall not contain more than 2 g of gluten per day. To make the upper limit for gluten consumption understandable for study participants, total daily gluten intake is color labeled to the correspondent content in the most commonly consumed foods in infants and toddlers. Food items are labeled as green, yellow, or red, and the allowed daily intake is limited to consumption of one portion of food items marked as red or two portions of food items marked as yellow. Serum is collected every 6 months for the first 5 years and then annually up to the age of 10 years and analyzed for tTGA using radio binding assays. The primary study outcome is CDA, and the secondary outcome is biopsy proven celiac disease.

## Discussion

The first 1,000 days of life (i.e., from conception to the child’s second birthday) are often mentioned as the most important window for shaping the child’s immune system and microbiome (30). Furthermore, studies have suggested that the gut microbiota affects gluten digestion, intestinal permeability, and the host immune system (31). Vice versa, early infant diet shapes the microbiome, and gluten may modify the composition of the intestinal flora (30, 32, 33). Despite the fact that incidence of celiac disease peaks in early childhood and has been found to be associated with both higher gluten intake and frequent gastrointestinal viral infections in previous prospective observational studies (15, 34, 35), infant gluten intervention with either delayed gluten introduction or introduction to small amounts during the child’s first year of life have failed to protect genetic-susceptible children from celiac disease in clinical trials (19, 20, 22). Gluten infant feeding may have an impact on the gut microbiome composition and diversity and, if being deranged during the early phase of development, may lead to autoimmunity (18, 36). Therefore, more clinical trials on dietary intervention with reduced gluten, probiotics, or both need to be evaluated in genetically at-risk children if primary prevention of celiac disease is even possible. Moreover, larger randomized clinical trials (RCTs) with longer follow-up are therefore required to prove the causality of reduced gluten amounts introduced to the child’s diet, not only at the time of introduction but also in a longer time frame, preventing genetic at-risk individuals from lifelong celiac disease.

So far, an introduction of small amounts of gluten in a time window between 4 and 6 months of age, given to induce tolerization, did not prevent celiac disease. However, infant gluten interventions beyond this tight time window with longer intervals have not been fully evaluated. However, recent publications with longer follow-up in the PREVENT-CD cohort show contradictory results (37–39). A study based on the Italian study population found that children who developed celiac disease by the age of 6 years had significantly higher gluten consumption after 24 months of age compared with children who did not develop celiac disease (39). Results from clinical trials testing these hypotheses are still pending. It is therefore too early to rule out that gluten intervention strategies may or may not be efficient targets of primary prevention of celiac disease in genetically at-risk children.

Peak incidence of seroconversion to celiac disease occurs early (15), suggesting that possible prevention strategies should be initiated probably before the age of 3 years. Prospective studies have shown that a high gluten intake compared to a low gluten intake during the second year of life is associated with increased risk of celiac disease (15–17, 39). Especially, in a multinational pediatric at-risk population, it was shown that daily gluten intake was associated with higher increased risk of developing CDA and with celiac disease for every 1-g/day increase in gluten intake, and a daily gluten intake above 2 g/day almost doubled the risk of developing celiac disease by the age of 3 years (15). These observational prospective studies suggest that preventive strategies should be targeted not only for a short time window during the introduction of gluten and weaning but maybe also for a longer period and up to 2 years of age at least. The two ongoing clinical trials (PreCiSe and GRAIn) have adopted this approach of a late gluten introduction or restricted gluten consumption, but it will take several years with longitudinal follow-up to find out if a delayed full gluten consumption to age 3 or 5 years will prevent celiac disease or only postpone the diagnosis to older ages.

It is hypothesized that exposures to viral infections while inducing tolerance to gluten may modify the risk of celiac disease. The steep increase in amount of gluten consumption during the first years of life coincides with a period with repeated virus infections (35). A previous study showed that children who later developed CDA had been more exposed to rotavirus infections (40). Later, the TEDDY study demonstrated that rotavirus vaccination protected children from CDA, but only in children that were introduced to gluten before 6 months of age (41). Many countries have implemented rotavirus vaccination into their national vaccination programs during the past decades. In Sweden, which has one of the highest prevalence of celiac disease in the world, reaching almost 3% (42), two rotavirus vaccines that are FDA approved (Rotarix^®^ and RotaTeq^®^) are currently given to all newborn babies born after the first of September, 2019. Only in a few years, it will be possible to perform a nationwide registry study to see if the national rotavirus vaccination program has an incipient effect on celiac disease incidence.

Another study from TEDDY showed that frequent exposures of enterovirus B during the first 2 years of life was associated with increased risk of CDA (41) and that higher amounts of gluten intake confer a cumulative effect on the risk of developing celiac disease autoimmunity after an enterovirus B infection, suggesting an interaction between infections and gluten in the etiology of celiac disease (35). The main findings from these studies showing complex interactions between gluten and virus infections support the notion that frequent viral infections may act as triggers, whereas gluten drives the inflammation in celiac disease. The mechanisms of why a potential viral infection would trigger an immune activation of T cells against gluten peptides and activation of B cells to produce tTGA in celiac disease are not fully understood. Although a potential causal role of virus and gluten in celiac disease pathogenesis remains to be confirmed, no RCT has yet studied if excluding gluten in young children leads to a lower incidence of celiac disease.

Although many baby formulas are fortified with probiotics to maintain the intestinal barrier by preventing crossing of immunogenic polypeptides to the lamina propria, few studies have evaluated their potential protective effects on celiac disease. Probiotics are live microorganisms that have demonstrated beneficial effects on human health after being administered in adequate amounts (43) and is considered promising additional treatments for various intestinal diseases by contributing to the stability of the gut microbiome and regulation of innate and adaptive immune systems (31, 44). A change in the gut microbiota homeostasis may eventually lead to dysbiosis and hypothetically contribute to the pathogenesis of autoimmune diseases (31). There are some indications showing that a combination of two *Lactobacillus* strains have dampening effects on the peripheral immune response in smaller studies on children with CDA (24). Probiotics may therefore have potential anti-autoinflammatory effects by promoting a healthy gut. Larger clinical trials on the use probiotics to prevent celiac disease are scarce, and it needs to be further evaluated if probiotics should be included in the arsenal of preventive measures of celiac disease. We have therefore extended our previous findings on *Lactobacillus*, which have proven effects on the gut permeability and positive immunoregulatory functions (24), to test if these two strains could act favorably on the gut mucosa layer. In PreCiSe, children will be randomized to either GFD, *Lactobacillus*, or placebo for the first 3 years of life, the period in life when children are being exposed and develop immunization to many common viruses. The study is ongoing and will be the first clinical trial to compare if interventions with probiotics will prevent children from having celiac disease as compared with children on a GFD or normal diet.

In conclusion, there are credible evidence from prospective studies that a higher intake of gluten in early childhood is associated with higher incidences of celiac disease. There are indications of a viral trigger in celiac disease, but no study has proven the causality of a virus in the celiac disease etiology, although prospective studies show interactions between gluten amount and frequent virus infections and subsequent risk of CDA. A cost-effective, safe, and tolerable prevention method of celiac disease has been long searched for. So far, no clinical trial has yet proven that an early gluten intervention is fully successful in preventing celiac disease. Several clinical trials are ongoing to evaluate long-term preventive effects of late gluten introduction in the diet and probiotics after the child’s immune system is fully developed by age 3. These longitudinal studies are pending and will take many years before being completed.

## Author contributions

CA drafted the manuscript. CA and DA both contributed and wrote the review. Both authors approved the submitted version.
